# Developmental, Behavioral and Transcriptomic Changes in Zebrafish Embryos after Smoke Dye Exposure

**DOI:** 10.3390/toxics10050210

**Published:** 2022-04-22

**Authors:** Edward J. Perkins, Kimberly T. To, Lindsey St. Mary, Charles H. Laber, Anthony J. Bednar, Lisa Truong, Robyn L. Tanguay, Natàlia Garcia-Reyero

**Affiliations:** 1 Environmental Laboratory, US Army Engineer Research & Development Center, Vicksburg, MS 39180, USA; edward.j.perkins@erdc.dren.mil (E.J.P.); kimberly.t.to@erdc.dren.mil (K.T.T.); charles.h.laber@usace.army.mil (C.H.L.); anthony.j.bednar@usace.army.mil (A.J.B.); 2 Sinnhuber Aquatic Research Laboratory, Department of Environmental and Molecular Toxicology, Oregon State University, Corvallis, OR 97333, USA; stmarylindsey@gmail.com (L.S.M.); lisa.truong@oregonstate.edu (L.T.); robyn.tanguay@oregonstate.edu (R.L.T.)

**Keywords:** transcriptomics, pathway, zebrafish embryo test, behavior, smoke dye

## Abstract

(1) Background: Disperse Blue 14, Disperse Red 9, Solvent Red 169 and Solvent Yellow 33 have been used to color smoke; however, they have not been comprehensively assessed for their potential health hazards. (2) Methods: To assess the effects of these dyes, zebrafish embryos were exposed from 6 to 120 h post fertilization (hpf) to 10–55 µM Disperse Red 9, 1–50 µM Solvent Red 169, 7.5–13.5 µM Solvent Yellow 33 or 133–314 µM Disperse Blue 14. Embryos were monitored for adverse effects on gene expression at 48 hpf as well as for mortality, development and behavior at 120 hpf. The dyes were examined for their potential to cross the blood–brain barrier. (3) Results: Solvent Yellow 33 and Disperse Blue 14 impaired development and behavior at all concentrations. Disperse Red 9 impaired behavior at all concentrations and development at all concentrations except for 10 µM. Solvent Red 169 caused no effects. Mortality was only seen in Disperse Blue 14 at 261.5 and 314 µM. Gene expression indicated impacts on neurodevelopment and folate and retinol metabolism as potential mechanisms of toxicity. (4) Conclusions: Smoke dyes have a high potential for causing developmental changes and neurotoxicity and should be examined more closely using comprehensive approaches as used here.

## 1. Introduction

Synthetic anthraquinone and quinoline dyes are used for a wide range of applications including colored smoke for entertainment, safety or military purposes and in consumer products including paper, plastics, leather, cosmetics, food, and the textile industry [1]. Anthraquinones are a major portion of dyes produced and the dye industry is responsible for as much as 20% of industrial water pollution annually [2,3,4,5]. Because of the presence of dyes in consumer products and wastewater, resistance to degradation and a relatively low level of toxicity data, significant concern has been raised regarding their discharge into the environment [2].

Toxicological information on dyes is limited in these broad classes of chemicals. This is especially true for dyes used in smokes and obscurants which pose a potential inhalation and dermal exposure risk to users in addition to ecological risks as a result of production and use in the environment. While data for certain dyes used in smoke formulations enable users to understand the potential hazards that the dyes pose, others have little information. For example, the quinoline dye Solvent Yellow 33 (also known as D&C yellow No 11), 2-(2-Quinolyl)-1,3-indandione, is a component of green and yellow dye mixtures for smoke coloring that is also used in pesticide products, lacquers, plastics, topical drugs and cosmetics [6]. Toxicological evidence indicates that Solvent Yellow 33 can cause contact dermatitis in some people [6,7,8]. A two-year study of F344/N rats continuously exposed to Solvent Yellow 33 in their feed indicated some evidence of carcinogenic activity in male and female rats [9]. Studies in human HepG2 cells indicated that Solvent Yellow 33 may cause DNA damage by modulating genes involved in DNA repair [10]. Tarnow et al. [11] identified Solvent Yellow 33 as a potential disruptor of Aryl Hydrocarbon Receptor/Estrogen Receptor signaling, which could have toxicological implications. Perinatal toxicity studies of F344/N rats exposed to 0, 5, 17 or 50 g/kg Solvent Yellow 33 revealed no reproductive and developmental effects as a result of exposure [12]. This level of information is sufficient to enable some assessment of health risks due to exposures to Solvent Yellow 33.

Unfortunately, many dyes used in smokes and other applications have little hazard information. For example, Disperse Red [9,1-(methylamino)anthraquinone], used in red- and violet-colored smokes and dye formulations (NRC 1999), has been found to be a skin irritant and sensitizer in humans but little evidence for toxic effects has been found [6]. Solvent Red 169 [1-(Isopropylamino) anthraquinone], used in dye formulations for coloring fabrics, ink, food, plastics and making violet smoke, have no data available for toxicity [13]. Disperse Blue 14 [1,4-bis(methylamino) anthracene-9,10-dione] has been used in dye formulations to make violet smokes and fireworks in addition to color fabrics and lubricants [3,14]. Disperse Blue 14 has been found to cause behavioral and developmental effects in zebrafish embryos [15].

Recently, Dilger et al. [16] examined the breakdown of Disperse Red 9 in a mock red flare and found that it pyrolyzed into many potentially toxic compounds including chloro(methylamino) anthraquinone. Machine learning quantitative structure–activity relationship models were then applied to predict several toxicological endpoints for Disperse Red 9 and its chlorinated derivative along with several other anthraquinone dyes and their chlorinated by products. While empirical data are not available, modeling predictions suggest that anthraquinone dyes are more toxic than previously thought, especially with respect to developmental toxicity.

To better characterize the potential toxicity of Disperse Red 9 and the dyes Disperse Blue 14, Solvent Red 169 and Solvent Yellow 33, we examined the effects of these dyes on zebrafish embryo development and function. Those specific dyes were chosen as they are used in colored smokes and there was a lack of hazard information on them. Zebrafish embryo exposures were combined with gene expression and in vitro blood–brain barrier permeability analysis to develop a comprehensive impact assessment. 

## 2. Materials and Methods

### 2.1. Analytical Chemistry 

The smoke dyes Disperse Blue 14 (DB14; CAS 2475-44-7), Solvent Red 169 (SR169; CAS 27354-18-3), Solvent Yellow 33 (SY33; CAS 8003-22-3) and Disperse Red 9 (DR9 CAS 82-38-2) were obtained from Walrus Enterprises LLC (Northampton, MA, USA). Samples were dissolved in dimethyl sulfoxide (DMSO) for animal exposures and then further diluted in methylene chloride and analyzed by an Agilent 6890 GC–MS using a poly (5% diphenyl/95%dimethylsiloxane) column measuring 30 m × 0.25 mm × 0.25 um to determine exposure concentrations. The oven parameters were as follows: initial temperature 40 °C held for 0.5 min, 10 °C/min to 100 °C, 25 °C/min to 280 °C held for 3 min, 5 °C/min to 300 °C held for 3 min, 25 °C/min to 325 °C. The analysis was performed following a modified SW 846 Method 8270 utilizing a 5-point calibration curve and internal standards. Data calculations were performed using Chemstation (Agilent Technologies, Santa Clara, CA, USA). See Table 1 for summarized results of analytical chemistry. The measured concentrations were between 33.8 and 56.6% of the nominal ones.

### 2.2. Zebrafish Husbandry 

Tropical 5D wild-type zebrafish were housed at the Oregon State University Sinnhuber Aquatic Research Laboratory. Adult fish were fed twice daily with Gemma Micro 500 (Skretting; Westbrook, ME, USA). The recirculating system was maintained at 28 °C with a 14 h light, 10 h dark cycle. The fish were kept at a density of 300 fish per 50 gallon tank filled with system water supplemented with Instant Ocean salt (Spectrum Brands; Blacksburg, VA, USA). The night prior, a spawning funnel was placed into the tanks. In the morning, embryos were collected and placed into embryo medium containing 15 mM NaCl, 0.5 mM KCl, 1 mM MgSO_4_, 0.15 mM KH_2_PO_4_, 0.05 mM Na_2_HPO_4_, and 0.7 mM NaHCO_3_ and kept at 28 °C in an incubator. 

### 2.3. Exposures 

Fertilized embryos were selected and staged according to Kimmel et al. [17]. The chorions of 4 h post fertilization (hpf) embryos were removed using 83 µL of 25.3 U/µL pronase (Roche, Indianapolis, IN, USA) using a custom automated dechorionator as described in Mandrell et al. [18]. At 6 hpf, the embryos were transferred into individual wells of a round-bottom 96-well plate filled with 100 µL of embryo medium. The smoke dyes were dispensed into each well using a HP D300 Digital dispenser. Each dye was suspended in 100% DMSO and added to the single-use cassette wells of the D300 at 20 mM. After the dyes were dispensed, the wells were normalized with 0.64% DMSO. Solvent Red 169 was tested at 0, 1, 5, 11.2, 35.6, and 50 µM; Solvent Yellow 33 was tested at 0, 7.5, 9, 10.5, 12 and 13.5 µM; Disperse Red 9 was tested at 0, 10, 25, 45, and 55 µM and Disperse Blue was tested at 0, 113, 171, 209, 261.5, and 314 µM. The concentrations were selected from a dose range-finding study that identified the maximal tolerable concentration and then a log 1/3 dilution series (Solvent Red, Solvent Yellow 33, and Disperse Blue). Disperse Red 9 test concentrations were determined based on a target concentration of 25 μM. For each of the dyes, embryos were exposed to 6 concentrations with 32 animals per concentration on two replicate plates. Afterwards, parafilm was placed between the lid and the wells to reduce evaporation. The plates were placed on an orbital shaker at 235 rpm at 28 °C for 16 h to create a homogenous test solution before being placed in a static incubator until 120 hpf. It is worth noting that while these exposures were performed under very controlled and robust conditions, abiotic environmental factors can affect the sensitivity of fish eggs in less controlled environments. 

### 2.4. Developmental Toxicity Assessments 

After the chemical exposures were initiated, the embryos were kept in the dark. At 24 hpf, embryos were assessed for mortality or delays in development. The embryos were placed back into the incubator and then kept in the dark until 120 hpf. At 120 hpf, dead animals were removed and embryos were subjected to a larval photomotor response assay (LPR) using the Viewpoint Zebrabox system (Viewpoint Life Sciences, Lyon, France). The assay was a total of 24 min, with a datapoint recorded every 6 s. The total distance was tracked for each of the 4 light–dark cycles, with the first 1 cycle treated as an acclimation period and discarded from analysis. Each cycle consisted of 3 min of alternating visible light (1000 lux) and dark (IR). Animals exhibiting morbidity or mortality were excluded from the analysis. After LPR, the embryos were assessed for a suite of malformations [19] which included yolk sac or pericardial edema, bent body axis, trunk length, caudal and pectoral fin, pigmentation, somite deformities, eye, snout, jaw, otolith malformations, gross brain development, notochord and circulating deformities, swim bladder presence and inflation, and the presence of a touch response. These effects were collected in a binary manner and stored in a laboratory information management system [19]. Developmental Lowest Observable Effect Level (LOEL) was identified as the lowest concentration level eliciting a significant difference from control.

### 2.5. Blood–Brain Barrier Permeability

The ability of dyes to pass through the blood–brain barrier was determined using a blood–brain barrier parallel artificial membrane permeability assay (BBB-PAMPA). BBB-PAMPA were performed by Creative Bioarray (Shirley, NY, USA) as described in Rabal et al. [20] and To et al. [15]. All compounds were tested with an incubation time of 4 h and at a concentration of 10 µM with propranolol as the positive control. Briefly, the donor solutions of smoke dye (10 μM, 150 μL in phosphate-buffered saline (PBS)/DMSO 19:1) were added to each well of the donor plate, whose polyvinylidene fluoride membrane was precoated with 5 μL of 1% brain polar extract (porcine)/dodecane mixture. Then, 300 μL of PBS was added to each well of the polytetrafluoroethylene acceptor plate. The donor and acceptor plates were combined and incubated for 4 h at room temperature with shaking at 300 rpm. In each plate, dyes and the positive control were tested in duplicate. After incubation, acceptor samples were prepared by mixing 270 μL of the solution from each acceptor well with 130 μL of acetonitrile containing the internal standard. Donor samples were prepared by mixing 20 μL of the solution from each donor well with 250 μL of PBS and 130 μL of acetonitrile containing the internal standard. Then, dye concentrations in the acceptor and the donor wells were analyzed by LC–MS/MS. The permeability rate (*Pe* in nm/s) was calculated with the following equation:

*Pe* = *C* × (−ln(1 − [drug]_acceptor_/[drug]_equilibrium_)) × 10^7^ (1)

where C = (VD × VA/((VD + VA) × area × time));

[*drug*]equilibrium = ([*drug*]donor × VD + [*drug*]acceptor × VA)/(VD + VA);
[*drug*]acceptor = (Aa/Ai × DF)acceptor;
[*drug*]donor = (Aa/Ai × DF)donor;
VD = 0.15 mL; VA = 0.30 mL; *area* = 0.28 cm^2^; *time* = 14,400 s;
Aa/Ai: Peak area ratio of NAC and the internal standard; DF: Dilution factor (13.5).

Finally, the permeability of the tested compounds was classified by their *Pe* as high (*Pe* > 10 nm/s), moderate (1 < *Pe* < 10), or low (*Pe* < 1).

### 2.6. Statistical Analysis of Toxicity Endpoints 

Statistical analyses were conducted in R v.3.6.1 [21]. Morphological endpoints were compared between the treatment groups and the control group using Fisher’s Exact Test. Significance was defined by the Bonferroni-adjusted *p*-value (0.05/5), which was adjusted for the concentration within each dye. Behavior was analyzed separately for the light phase and the dark phase. Within each phase, the distribution of average movement per fish was compared between the treatment and control using the Kolmogorov–Smirnov test. Significance was defined by the Bonferroni-adjusted *p*-value (0.05/5), which was adjusted for the concentration within each dye. Behavioral LOELs were identified as the lowest concentration level eliciting a significant difference from control.

### 2.7. RNA Sequencing 

At 48 h, 4 exposed embryos from each treatment were removed for RNA sequencing. RNA-Seq libraries for each embryo were prepared using 200 ng of total RNA and the NEBNext^®^ Ultra™ II RNA Library Prep (NEB, Cat: E7765S) per the manufacturer’s instructions, with mRNA enriched via poly-A-selection using oligoDT beads. The RNA was then thermally fragmented and converted to cDNA, adenylated for adaptor ligation and PCR amplified. The libraries were sequenced using the NovaSeq 6000 with 150 bp paired-end reads. RTA (version 2.4.11; Illumina, San Diego, CA, USA) was used for base calling and analysis was completed using MultiQC v1.7 (https://multiqc.info/).

### 2.8. Analysis of RNA-Seq Data

Raw RNA-Seq reads were trimmed of adaptors and low-quality sequences with Trim Galore v.0.6.5 (https://www.bioinformatics.babraham.ac.uk/projects/trim_galore/) using default settings for Illumina paired-end reads. Read quality was evaluated from the MultiQC report generated within Trim Galore (Supplemental File S1). Trimmed reads were quantified using Salmon v.1.1.0 with the default settings for paired-ends reads in selective alignment mode via the –validateMappings option (Supplemental File S2). Reads were aligned to a decoy-aware transcriptome that was built using Ensembl release 97 and provided by the COMBINE-lab (http://bit.ly/30yn3FJ, accessed on 2 July 2020, Supplemental File S3).

Transcript quantification files were prepared for analysis by following the Bioconductor vignette for preparation of Salmon quantification files for edgeR (https://bioconductor.org/packages/release/bioc/vignettes/tximport/inst/doc/tximport.html). Briefly, quantification files were imported using tximport v.1.1.4.2 and mapped to Ensembl Gene IDs using the Bioconductor genome-wide annotation for zebrafish package org.Dr.eg.db v.3.10.0. Gene counts were normalized for gene length and effective library size. 

Transcript abundance files were prepared for analysis using tximport v.1.14.2 in R and mapped to Ensembl Gene IDs for gene-level inference. Gene counts were normalized for gene lengths and effective library size. Differential gene expression analysis was performed using edgeR v.3.28.1 in R. Briefly, for each dye, read counts were fit to a quasi-likelihood negative binomial generalized linear model with concentration groups as the dependent variable. Each concentration group was compared to its respective control using the empirical Bayes quasi-likelihood F test. Results from this test were filtered such that genes with a fold change greater than 1.5 and a false discovery rate less than 0.05 were considered differentially expressed. One sample was removed from the 12 μM Solvent Yellow 33 treatment group after QC.

Genes identified as differentially expressed in any treatment group were selected for further analysis. Enrichment analysis was performed for each treatment group using topGO v.2.38.1 and the kegga function from limma v.3.42.2 in R. GO terms and KEGG pathways were filtered such that those with a *p*-value < 0.01 and at least 5% of genes within the treatment group were within the term’s annotated gene list were considered enriched. For comparative analysis, genes that were differentially expressed in any treatment group were grouped using K-means clustering on the log_2_ (fold-change) values for each treatment. Transcriptional LOELs were identified as the lowest concentration eliciting a significant difference from control. 

## 3. Results

### 3.1. Mortality

**Figure 1:**
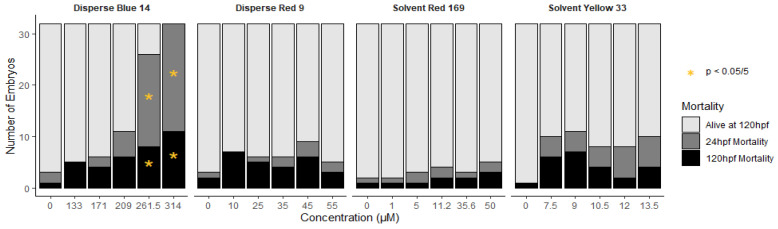
Summary of mortality at 24 and 120 hpf in Disperse Blue 14, Disperse Red 9, Solvent Red 169 and Solvent Yellow 33 treatment groups. * Indicates statistical significance (*p* < 0.05/5).

Of the four dyes, only Disperse Blue 14 caused a significant increase in mortality as early as 24 hpf. Significant mortality at both 24 and 120 hpf was only observed in embryos exposed to 261 and 314 µM treatment groups (Figure 1). It should be noted the concentration range of Disperse Blue 14 tested here (133–314 µM) was used to better understand toxicity since the dye had little effect on zebrafish embryos at 0.56–28.3 µM [15].

### 3.2. Morphological Effects

**Figure 2:**
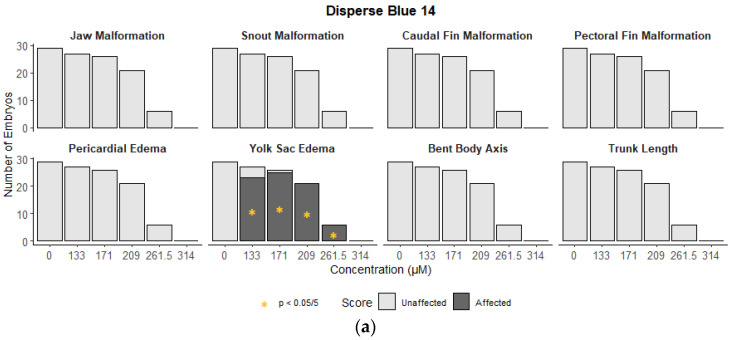

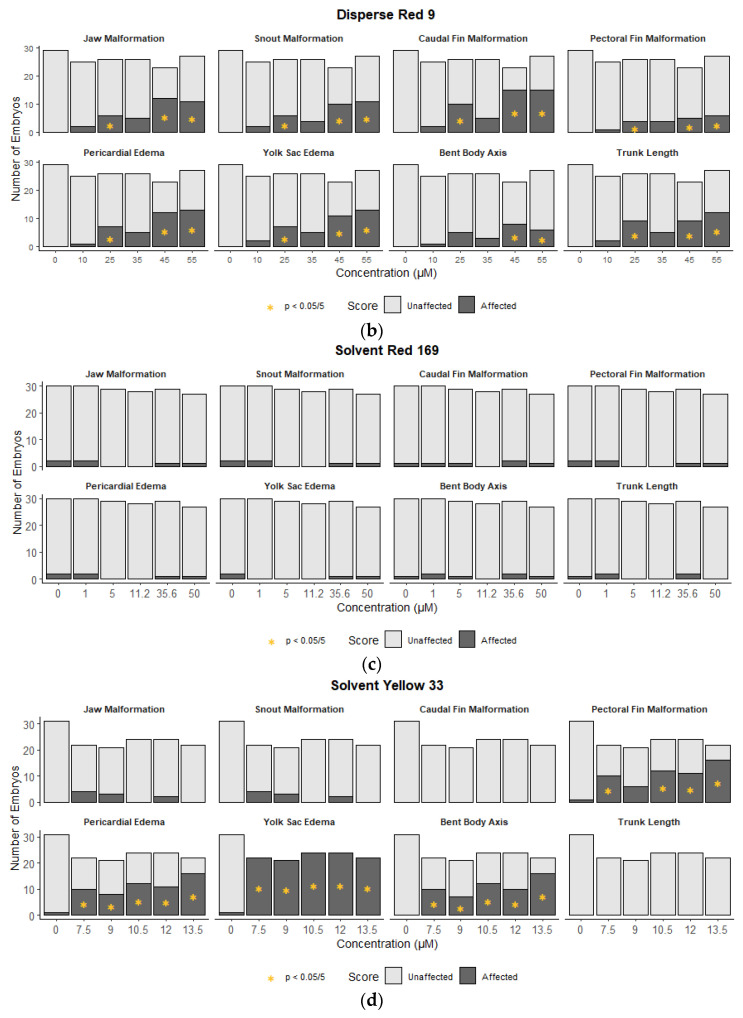
Summary of affected morphological endpoints in the Disperse Blue 14 (**a**), Disperse Red 9 (**b**), Solvent Red 169 (**c**), and Solvent Yellow 33 (**d**) treatment groups. * Indicates statistical significance (*p* < 0.05/5). The endpoints where no effects were seen are not included.

Disperse Blue 14 caused yolk sac edema at all levels tested, but was not significant for any other morphological endpoints (Figure 2a). Disperse Red 9 caused morphological effects in all eight categories examined with a LOEL of 25 µM for yolk sac edema, pericardial edema, trunk, snout, jaw, and caudal fin abnormalities; a LOEL of 45 µM for bent body axis abnormalities; and an LOEL of 55 µM for pectoral fin morphological effects (Figure 2b). Solvent Red 169 did not cause significantly higher morphological abnormalities than control exposures at any concentration (Figure 2c). Solvent Yellow 33 caused abnormalities at all concentrations tested for four of eight categories scored (yolk sac edema, pericardial edema, bent body axis, and pectoral fin) (Figure 2d). No effects were seen in the eye, otolith malformations, gross brain development, notochord and circulating deformities, swim bladder presence and inflation, nor the presence of a touch response (data not shown).

### 3.3. Effect of Dyes on Behavior

**Figure 3:**
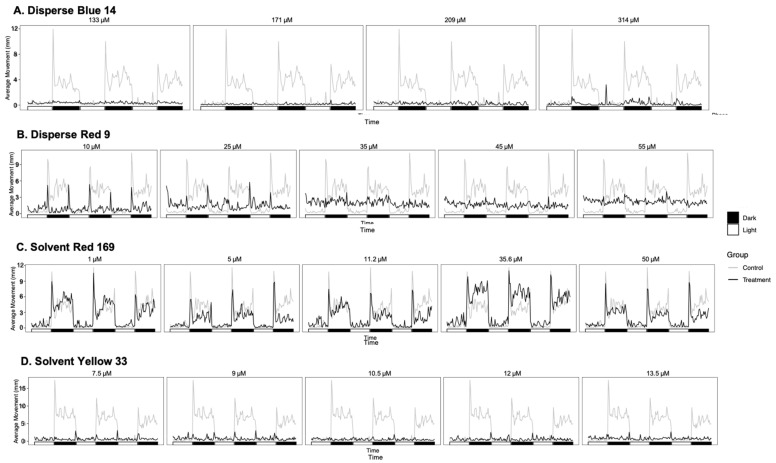
Average movement of the (**A**) Disperse Blue 14, (**B**) Disperse Red 9, (**C**) Solvent Red 169 and (**D**) Solvent Yellow 33 treatment groups throughout the photomotor assay.

We examined the effect of dyes on the zebrafish embryo developing nervous system using the larval photomotor response assay to monitor behavioral responses to light and dark (Figure 3, Supplemental Tables S1 and S2). Disperse Blue 14 had no effect on movement during the light phase. However, Disperse Blue 14 dramatically reduced the dark-phase movement (hypoactive) at all concentrations tested in groups having sufficient surviving embryos to test movement. Disperse Red 9 caused significantly higher movement (hypoactive) at all exposures above 10 μM in the light phase, but caused significantly lower dark-phase movement at 10 and 25 μM Disperse Red 9. Solvent Red 169 caused significantly higher movement than controls in the light phase (hyperactive) only at the second highest treatment group of 35.6 μM. Solvent Red 169 treatment had no effect on dark-phase movement. Solvent Yellow 33 had no significant effect on light-phase movement, but did dramatically lower dark-phase movement in all treatment groups (Figure 3).

The lack of movement in the dark phase observed with Disperse Blue 14 and Solvent Yellow 33 may be due to physical defects, rather than an indication of neurotoxicity [22]. Some textile dyes have been found to impair swim bladder inflation and function [23,24]. However, no significant changes in swim bladder function were observed in any treatment group (Table 2). In other studies, exposure of zebrafish embryos to the dyes erythrostominone and Solvent Violet 47 decreased dark-phase movement but did not decrease swim bladder function; therefore, the hypoactivity was attributed to yolk sac edema and pericardial edema [15,25]. Here, all treatment groups of Disperse Blue 14 displayed yolk sac edema and all concentrations of Solvent Yellow 33 displayed yolk sac and pericardial edema formation, which could therefore be a physical obstruction inhibiting swim ability. Disperse Red 9 treatments also caused yolk sac and pericardial edemas, albeit to a lower degree in each treatment group. However, Disperse Red 9 treatments displayed hyperactivity in all but the 10 μM treatment groups in the light phase and hypoactivity in the dark phase in the lowest treatment groups (10 and 25 μM). As the presence of edemas does not appear to directly correlate with loss of activity, Disperse Red 9 is likely to impair behavior through both physical and neurological effects.

### 3.4. Blood–Brain Barrier Permeability

The blood–brain barrier (BBB) is a physical barrier formed by tight junctions of endothelial cells of blood vessels in the brain. The barrier is highly selective and excludes many substances from entering the brain, thus protecting the brain against toxic chemical substances [26]. Chemicals that can cross the BBB could potentially compromise the central nervous system, and lead to neurotoxicity. Here, we tested the ability of Disperse Red 9, Disperse Blue 14 and Solvent Yellow 33 to cross the BBB in order to better understand their potential to induce neurotoxicity and impact neurodevelopment. Artificial membranes simulating the blood–brain barrier were highly permeable to Disperse Red 9, Disperse Blue 14 and Solvent Yellow 33, indicating that these dyes would readily cross the BBB (Table 3). Solvent Red 169 was not tested due to its low solubility in the assay buffer. The positive control propranolol was tested twice, both times providing a high permeability (mean Pe: 32.4 and 66.8 nm/s; mean recovery: 39.3 and 40.8%). This indicates that the dyes are able to enter the developing zebrafish brain to cause neurotoxic effects and is consistent with the dyes causing developmental impacts, behavioral impacts and impacts on gene expression of neurological development and sensory processes.

### 3.5. Disperse Blue 14 Differentially Expressed Genes and Functional Enrichment

**Figure 4:**
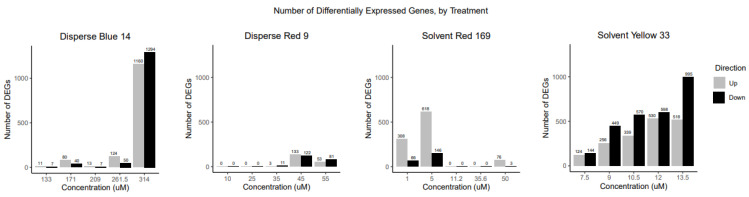
Number of up- and downregulated differentially expressed genes for the Disperse Blue 14, Disperse Red 9, Solvent Red 169 and Solvent Yellow 33 treatment groups.

At 48 hpf, Disperse Blue 14 concentrations below 314 μM caused relatively few DEGs (18–174) in comparison to 314 μM (2454 DEG; Figure 4), with the identity of most DEGs overlapping with those from the 314 μM treatment group (Supplemental Table S5). Stress-related impacts on gene expression were seen in the 171 and 261.5 μM treatment groups with enrichment and upregulation of the KEGG path:dre04115 p53 signaling pathway (5 up, 0 down; 6 up, 0 down at 171 and 261.5 μM), GO biological processes GO:0030018 inflammatory response (6 up, 0 down; 8 up, 0 down at 171 and 261.5 μM) and GO:0007050 cell cycle arrest (2 up, 0 down; 3 up, 0 down at 171 and 261.5 μM) (Supplemental Tables S6 and S7).

Consistent with the high mortality experienced at 314 μM of Disperse Blue 14, DEGs of GO:0000278 mitotic cell cycle were principally downregulated (19 up, 29 down) and GO:0009611 response to wounding was upregulated (23 up, 8 down) (Supplemental Tables S6 and S7). Additionally, consistent with increased mortality observed at the high dose, at 48 hpf, embryo KEGG pathways related to metabolism were downregulated [e.g., path:dre01100 metabolic pathways (78 up, 156 down) and path:dre00190 oxidative phosphorylation (0 up, 36 down)]. Pathways were also affected that were related to protein misfolding [path:dre04141 protein processing in endoplasmic reticulum (10 up, 22 down)], cellular damage and senescence [path:dre04218 cellular senescence (21 up, 13 down), path:dre04140 autophagy-animal (22 up, 9 down)] and DNA replication and repair [path:dre03030 DNA replication (0 up, 10 down), path:dre03420 nucleotide excision repair (2 up, 10 down) and path:dre03430 mismatch repair (0 up, 7 down)].

Notably, few GO terms or KEGG pathways related to neuronal processes were enriched in doses below 314 μM with the exception of GO:0014004 microglia differentiation (2 up, 0 down) and GO:0048679 regulation of axon regeneration (2 up, 0 down) at 261.5 μM. This supports the hypothesis that dark phase hypoactivity seen in all treatment groups in the locomotor assay is likely due to physiological defects (yolk sac edema) rather than neurotoxicological effects. Yolk sac edema is a common endpoint that many chemicals can perturb. This is not to say that the mechanism that leads to this endpoint is always the same. It is unknown if the yolk sac edema is a primary or secondary manifestation to the exposure. We used the zebrafish model as a biosensor to indicate that exposure to the dyes disrupted the most sensitive life stage and then used secondary assays and transcriptomics to identify the pathways involved to result in a phenotype later in development.

### 3.6. Disperse Red 9 Differentially Expressed Genes and Functional Enrichment 

At 48 hpf, Disperse Red 9 treatments had low levels of DEG in comparison to other dye treatment groups (Figure 4). No effect on gene expression was seen in the 10 and 25 μM treatment groups. The 35 μM group had 14 DEGs, with most overlapping in identity with DEGs from the two higher concentration treatment groups (Supplemental Table S5). GO terms in DEG at 35 and 45 μM were enriched for biological processes in sodium and potassium ion homeostasis (Supplemental Table S8). At both 45 and 55 μM, DEG were enriched for biological processes GO:0010165 response to X-rays (3 up, 0 down; 2 up, 0 down at 45 and 55 μM) and the KEGG pathway path:dre03440 homologous recombination (4 up, 0 down; 3 up, 0 down at 45 and 55 μM) (Supplemental Tables S8 and S9). At 55 μM, DEG were downregulated for biological processes in nerve and eye development (GO:0002088 lens development in camera-type eye (0 up, 4 down), GO:0021545 cranial nerve development (2 up, 2 down), and GO:0005212 structural constituent of eye lens (0 up, 4 down)). Although no eye deformities were observed in exposed embryo, the downregulation of nerve and eye development processes is consistent with Disperse Red 9 causing changes in light/dark locomotor responses seen in Section 3.3.

### 3.7. Solvent Red 169 Differentially Expressed Genes and Functional Enrichment

At 48 hpf, Solvent Red 169 principally affected genes in the two lowest treatment groups, 1 μM (374 DEG) and 5 μM (764 DEG), where most DEGs were upregulated (926 up vs. 512 down; Figure 4). A small number of genes (79) were also affected at the 50 μM concentration Functional enrichment analysis indicated Solvent Red 169 impacted several biological pathways related to regulation of gene expression and development (Supplemental Tables S3 and S4). DEGs in the 1 μM treatment group were principally enriched in Gene Ontology (GO) terms for biological processes and functions related to cell cycle [GO:0007050 cell cycle arrest (5 up, 0 down)] and transcriptional and mRNA regulation [GO:0000122 Negative regulation of transcription by RNA polymerase II (9 up, 2 down)]. The Kyoto Encyclopedia of Genes and Genomes (KEGG) pathway dre04216 ferroptosis (3 up, 2 down) which is involved in cell death was also enriched at 1μM Solvent Red 169. 

The Solvent Red 169 5 μM treatment group was enriched for upregulated GO biological processes related to cell cycle [GO:0007050 cell cycle arrest (5 up, 0 down)], transcriptional regulation [GO:0006355 regulation of transcription, DNA-templated (83 up, 5 down)], and neuronal development [GO:0050767 regulation of neurogenesis (16 up, 2 down), GO:0021884 forebrain neuron development (3 up, 0 down) and GO:0048702 embryonic neurocranium morphogenesis (3 up, 1 down)]. At 50 μM, the GO biological processes GO:0021695 cerebellar cortex development (2 up, 0 down) and GO:0006749 glutathione metabolic process (2 up, 0 down) were enriched and upregulated. While a number of biological processes related to neurodevelopment were impacted and upregulated in exposed embryos, this does not appear to have had measurable impacts on the developmental and behavioral end points examined at 120 hpf. This suggests that detection of transcriptional changes due to chemical exposure does not necessarily lead to adverse effects and may reflect adaptive processes.

### 3.8. Solvent Yellow 33 Differentially Expressed Genes and Functional Enrichment 

After 48 h of exposure to Solvent Yellow 33, embryos showed a concentration-dependent increase in number of DEGs (Figure 4). There was a high degree of overlap (55–96%) in identity of differentially expressed genes between the Solvent Yellow 33 treatment groups (Supplemental Table S5). A number of KEGG pathways were enriched at all exposure concentrations that are related to increased chemical stress (path:dre0048 glutathione metabolism, path:dre00982 metabolism of xenobiotics by cytochrome p450, path:dre00982 drug metabolism-cytochrome P450 and path:dre00480 glutathione metabolism).

Chequer et al. [27] found that quinoline yellow (Solvent Yellow 33) can cause DNA damage to exposed human HepG2 cells in culture to nominal concentrations of 1.8, 3.6, 7.3, 18, 37, 55 and 73 μM and found that concentrations of 7.3 uM and higher were genotoxic and caused chromosomal damage using comet assays to detect genomic DNA degradation. Solvent Yellow 33 induced pathways involved in DNA damage and cell death in exposed embryos, suggesting that Solvent Yellow 33 may also be genotoxic to exposed embryos. The KEGG pathway path:dre04216 ferroptosis, an iron-dependent regulated form of cell death caused by the accumulation of lipid-based reactive oxygen species [28] was enriched in all but the 12 μM treatment group (3 up, 1 down, 5 up, 1 down; 5 up, 2 down; 9 up, 1 down at 7.5, 9, 10.5 and 13.5 μM, respectively; Supplemental Table S10). The KEGG path:dre04115 p53 signaling pathway which is induced by cell and DNA damage was upregulated in exposed embryos at 10.5 μM and above, (8 up, 1 down; 12 up, 0 down; 12 up 1 down at 10.5, 12 and 13.5 μM, respectively; Supplemental Table S10). 

Consistent with hypoactivity in dark-phase movement by exposed larva at 120 hpf (Figure 3), there was a decrease in expression of GO biological processes related to eye development/function in all treatment groups at 48 hpf [GO:0061074 regulation of neural retina development (0 up, 2 down; 1 up, 2 down and 1 up, 2 down at 7, 9 and 10.5 μM); GO:0071482 cellular response to light stimulus in (1 up, 6 down; 2 up, 7 down; 2 up, 6 down and 1 up, 8 down at 9, 10.5, 12, 13.5 μM) and GO:0007602 phototransduction (0 up, 5 down at 9; 0 up, 6 down; 0 up, 7 down at 9, 10.5, 13.5 μM) Supplemental Table S11)]. 

Solvent Yellow 33 reduced expression of metabolic pathways essential in embryo development including KEGG path:dre00790 folate synthesis (1 up, 4 down in all treatments), path: dre00360 phenylalanine metabolism (1 up, 2 down; 1 up, 3 down; 1 up, 3 down; 1 up and 3 down at 7.5, 9, 10.5 and 12 μM), path:dre00830 4etinol metabolism (4 up 8 down, 4 up, 6 down, 4 up 7 down at 9, 10.5 and 12 μM) (Supplemental Table S10). Disruption of folate synthesis causes developmental defects such as ventral edema, dorsal curvature, a shortened anterior–posterior axis and cardiac defects [29]. Disruption of retinoic acid signaling in the embryo has been found to cause deformations in pectoral fins [30] and impair eye development [31,32]. The effects of Solvent Yellow 33 exposure include *pericardial edema* (cardiac defects), bent body axis (dorsal curvature), pectoral fin deformation and downregulation of pathways related eye development/function, suggesting that disruption of folate synthesis and retinol metabolism may contribute to the developmental toxicity associated with Solvent Yellow 33.

## 4. Discussion

We examined the effect of three anthraquinone and one quinoline dye on the development, behavior and gene expression of zebrafish embryos to better understand the potential toxicity of the dyes. The combination of embryo testing with transcriptomics and in vitro assays for BBB permeability has allowed us to develop a more accurate understanding of the potential hazards of these dyes. The four dyes had a wide range of toxicity, with all but Solvent Red 169 causing adverse effects on development and behavior. 

Solvent Red 169 had the least impact, with no effect on mortality or morphology, a slight effect on behavior and moderate, mostly upregulated, effects on gene expression (0–764 DEG) at the range of concentrations tested (1–50 μM). Transcriptomics did not identify any potential negative impacts of Solvent Red 169. 

Disperse Red 9 had no effect on mortality at the 10–55 μM range tested, a LOEL of 25 μM for morphological abnormalities where it affected 8 of 13 categories, a LOEL of 25 μM for hyperactivity in light and hypoactivity in the dark phase of locomotor tests and a LOEL of 35 μM for a relatively low number of DEG (14–255 DEG per treatment). Gene expression analysis indicated that DNA damage and disruption of nerve and eye development may be occurring at the 55 μM dose tested at 48 hpf.

Disperse Blue 14 had a greater impact than Solvent Red 169 and Disperse Red 9 on the endpoints tested. Disperse Blue 14 had a LOEL of 261.5 μM for mortality, caused yolk sac edema and hypoactivity in the dark phase at all concentrations and had a LOEL of 133 μM for gene expression, with 314 μM affecting the most DEGs (2454 DEG vs. 18–174 for other treatment groups) at the 133–314 μM range tested. Gene expression analysis indicated that the dye caused toxic effects at all concentrations but few effects on neurodevelopmental processes. Our previous assessment of Disperse Blue 14 indicates that the dye causes developmental and behavioral effects at much lower concentrations, with a LOEL of 20.2 μM for developmental effects and hyperactivity in light and 0.66 μM for hypoactivity in dark [15].

While Solvent Yellow 33 had no effect on mortality, it was the most potent dye tested. It caused abnormalities in 4 of 13 categories at all concentrations tested (LOEL 7.5 μM), dark-phase hypoactivity at LOEL of 17.5 and affected a number of genes at each dose (268–1513 DEG) at the 7.5–13.5 μM range tested. At all concentrations, Solvent Yellow 33 decreased expression of metabolic pathways essential to embryo development (folate and retinol), whose disruption is known to cause developmental effects seen in exposed embryos.

Gene expression and enrichment analyses could also provide some insights into potential treatments. For instance, both Solvent Red 169 and Solvent Yellow 33 exposures affected glutathione metabolism pathways. Treatments that help replenish glutathione levels (such as N-acetylcysteine) could be used to validate or disprove the particular pathway as well as potentially be considered to counteract the adverse effects [33,34].

Anthraquinone and quinoline dyes are widely used across the world and potentially pose human and environmental hazards due to their use in commercial products and discharge into the environment. The analyses presented here indicate that certain dyes may have significant impacts on the development and behavior of zebrafish embryos. Since the developing zebrafish embryo is a model system to understand potential effects in mammalian systems, this indicates that these dyes also pose potential hazards to humans. This work demonstrates that the application of embryo testing with transcriptomics can deepen our understanding of the potential health hazard of dyes.

## Figures and Tables

**Table 1:** Analytical chemistry analysis showing nominal versus measured concentrations.

Name	Specifications	CAS Number	MW	Measured (mM)	Nominal (mM)	% of Nominal
Solvent Red 169		27354-18-3	265.31	13.53	40	33.8
Solvent Yellow 33	DOD-D-51485	8003-22-3	273.29	13.94	40	34.8
Disperse Blue 14	Def Std 68-58/2	2475-44-7	266.29	22.64	40	56.6
Disperse Red 9	Mil-D-3284	82-38-2	237.25	14.65	40	36.6

**Table 2:** Lowest Observable Effect Levels (LOEL) and Fisher’s Exact Test (P) *p*-values for morphological endpoints. (-) indicates no significance at any concentration. At 24 hpf, embryos were assessed for mortality (MO24), developmental progression (DP24), spontaneous movement (S24), and notochord distortion (NC24). Additionally, at 120 hpf, embryos were assessed for mortality (MORT), and morphological malformations including yolk sac edema (YSE), bent body axis (AXIS), eye (EYE), snout (SNOU), jaw (JAW), otic (OTIC), pericardial edema (PE), brain (BRAI), somite (SOMI), pectoral fin (PFIN), caudal fin (CFIN), circulation (CIRC), pigmentation (PIG), trunk length (TRUN), swim bladder (SWIM), notochord distortion (NC), and alterations in touch response (TR).

Endpoint	Solvent Red 169		Disperse Blue 14		Disperse Red 9		Solvent Yellow 33	
	LOEL (µM)	*p*	LOEL (µM)	*p*	LOEL (µM)	*p*	LOEL (µM)	*p*
MO24	-	-	261.5 μM	1.20 × 10^−5^	-	-	-	-
DP24	-	-	-	-	-	-	10.5	0.0031
SM24	-	-	-	-	-	-	-	-
NC24	-	-	-	-	-	-	-	-
MORT	-	-	261.5 μM	0.00013	-	-	-	-
YSE_	-	-	133 μM	5.50 × 10^−12^	25	0.0032	7.5	5.00 × 10^−14^
AXIS	-	-	-	-	45	0.00065	7.5	3.30 × 10^−5^
EYE_	-	-	-	-	-	-	-	-
SNOU	-	-	-	-	25	0.0079	-	-
JAW_	-	-	-	-	25	0.0079	-	-
OTIC	-	-	-	-	-	-	-	-
PE__	-	-	-	-	25	0.0032	7.5	0.00027
BRAI	-	-	-	-	-	-	-	-
SOMI	-	-	-	-	-	-	-	-
PFIN	-	-	-	-	55	0.0091	7.5	0.00027
CFIN	-	-	-	-	25	0.00018	-	-
PIG_	-	-	-	-	-	-	-	-
CIRC	-	-	-	-	-	-	-	-
TRUN	-	-	-	-	25	0.00049	-	-
SWIM	-	-	-	-	-	-	-	-
NC__	-	-	-	-	-	-	-	-
TR__	-	-	-	-	-	-	-	-

**Table 3:** Blood–brain barrier permeability assay results. Pe is permeability. Low permeability: Pe < 1 nm/s. Moderate permeability: 1 < Pe < 10 nm/s. High permeability: Pe > 10 nm/s. ^1^ Disperse Blue 14 data from To et al. [15]. NT is not tested due to low solubility.

Compound	Mean Pe (nm/s)	% Mean Recovery	Permeability
Disperse Red 9	30.891	4.7	High
Disperse Blue 14	26.259	11.5	High
Solvent Yellow 33	48.517	20	High
Solvent Red 169	NT		

## Data Availability

RNA-Seq data reported here have been deposited in NCBI’s Gene Expression Omnibus (GEO; https://www.ncbi.nlm.nih.gov/geo) and are accessible through the GEO Accession number GSE166302.

## Supplementary Materials

The following supporting information can be downloaded at: https://www.mdpi.com/article/10.3390/toxics10050210/s1, File S1. RNA-Seq MultiQC report generated within Trim Galore. File S2. Supporting file for quantification of trimmed RNA-Seq reads using Salmon v.1.1.0, File S3. Supporting file for alignment if RNA-Seq reads to a decoy-aware transcriptome. Table S1: Summary of light-phase movement for surviving embryos and results of the Kolmogorov–Smirnov test of movement between treatment and control. Bolded values indicate significant differences in average movement at the Bonferroni-adjusted threshold (0.05/5). Table S2: Summary of dark-phase movement for surviving embryos and results of the Kolmogorov–Smirnov test of movement between treatment and control. Bolded values indicate significant differences in average movement at the Bonferroni-adjusted threshold (0.05/5). Table S3: Summary of Gene Ontology (GO) terms enriched in differentially expressed genes in embryos exposed to Solvent Red 169 until 48 h post fertilization. BP = biological process, MF = molecular function and CC = cellular component. Table S4: Summary of Kyoto Encyclopedia of Genes and Genomes (KEGG) pathways enriched in differentially expressed genes in embryos exposed to Solvent Red 169 until 48 h post fertilization. Table S5: Number of differentially expressed genes shared between all treatment groups. Table S6: Summary of Gene Ontology (GO) terms enriched in differentially expressed genes in embryos exposed to Disperse Blue 14 until 48 h post fertilization. BP = biological process, MF = molecular function and CC = cellular component. Table S7: Summary of Kyoto Encyclopedia of Genes and Genomes (KEGG) pathways enriched in differentially expressed genes in embryos exposed to Disperse Blue 14 until 48 h post fertilization. Table S8: Summary of Gene Ontology (GO) terms enriched in differentially expressed genes in embryos exposed to Disperse Red 9 until 48 h post fertilization. BP = biological process, MF = molecular function and CC = cellular component. Table S9: Summary of Kyoto Encyclopedia of Genes and Genomes (KEGG) pathways enriched in differentially expressed genes in embryos exposed to Solvent Yellow 33 until 48 h post fertilization. Table S10: Summary of Gene Ontology (GO) terms enriched in differentially expressed genes in embryos exposed to Solvent Yellow 33 until 48 h post fertilization. BP = biological process, MF = molecular function and CC = cellular component. Table S11: Summary of Kyoto Encyclopedia of Genes and Genomes (KEGG) pathways enriched in differentially expressed genes in embryos exposed to Disperse Red 9 until 48 h post fertilization.
